# Predictors of Recurrence and Survival in Cancer Patients With Pericardial Effusion Requiring Pericardiocentesis

**DOI:** 10.3389/fcvm.2022.916325

**Published:** 2022-05-31

**Authors:** Talha Ahmed, Elie Mouhayar, Juhee Song, Efstratios Koutroumpakis, Nicolas L. Palaskas, Syed Wamique Yusuf, Juan Lopez-Mattei, Saamir A. Hassan, Peter Kim, Mehmet Cilingiroglu, Konstantinos Marmagkiolis, Ara A. Vaporciyan, Stephen Swisher, Anita Deswal, Cezar Iliescu

**Affiliations:** ^1^Department of Cardiology, The University of Texas Health Science Center at Houston, Houston, TX, United States; ^2^Department of Cardiology, The University of Texas MD Anderson Cancer Center, Houston, TX, United States; ^3^Department of Biostatistics, The University of Texas Health Science Center at Houston, Houston, TX, United States; ^4^Department of Thoracic and Cardiovascular Surgery, The University of Texas MD Anderson Cancer Center, Houston, TX, United States

**Keywords:** malignant pericardial effusion, recurrence, pericardiocentesis, survival, cancer patients

## Abstract

**Aim:**

This study investigated the factors predicting survival and the recurrence of pericardial effusion (PE) requiring pericardiocentesis (PCC) in patients with cancer.

**Materials and Methods:**

We analyzed the data of patients who underwent PCC for large PEs from 2010 to 2020 at The University of Texas MD Anderson Cancer Center. The time to the first recurrent PE requiring PCC was the interval from the index PCC with pericardial drain placement to first recurrent PE requiring drainage (either repeated PCC or a pericardial window). Univariate and multivariate Fine-Gray models accounting for the competing risk of death were used to identify predictors of recurrent PE requiring drainage. Cox regression models were used to identify predictors of death.

**Results:**

The study cohort included 418 patients with index PCC and pericardial drain placement, of whom 65 (16%) had recurrent PEs requiring drainage. The cumulative incidences of recurrent PE requiring drainage at 12 and 60 months were 15.0% and 15.6%, respectively. Younger age, anti-inflammatory medication use, and solid tumors were associated with an increased risk of recurrence of PE requiring drainage, and that echocardiographic evidence of tamponade at presentation and receipt of immunotherapy were associated with a decreased risk of recurrence. Factors predicting poor survival included older age, malignant effusion on cytology, non-use of anti-inflammatory agents, non-lymphoma cancers and primary lung cancer.

**Conclusion:**

Among cancer patients with large PEs requiring drainage, young patients with solid tumors were more likely to experience recurrence, while elderly patients and those with lung cancer, malignant PE cytology, and non-use of anti-inflammatory agents showed worse survival.

## Introduction

Pericardial effusion (PE) is relatively common in cancer patients and is primarily caused by tumor invasion or disease treatment (1). Among cancer patients, malignant PE frequently occurs in those with advanced disease and is associated with worse outcomes (2, 3). The spectrum of malignant pericardial disease ranges from asymptomatic PE to hemodynamic instability in the setting of cardiac tamponade or constrictive physiology. Despite aggressive treatment, the prognosis of cancer patients with PE remains poor and is primarily dictated by the characteristics of the underlying disease (4). The treatment of PE attempts to correct hemodynamic instability and minimize interruptions in cancer therapy with the long-term goal to prevent effusion recurrence.

It is unknown which method of managing PE with imminent or recurrent tamponade is the most effective; however, pericardiocentesis (PCC) and surgical drainage (*via* a pericardiotomy or pericardial window) are widely used (5). The management of patients with PE and tamponade should be determined by the probability of recurrence of PE and expected survival time. Little information exists regarding the factors that may predict development of recurrent PE in these patients. The duration from the index PCC to first recurrence of PE requiring another drainage is also not well studied. Furthermore, the impact that recurrent PE has on the treatment and overall prognosis of cancer patients with PE is not known. Therefore, the aim of this study was to identify the factors predicting survival and recurrent PE requiring PCC in cancer patients.

## Materials and Methods

We conducted a retrospective analysis of a cohort of cancer patients who underwent index PCC from 2010 to 2020 at The University of Texas MD Anderson Cancer Center and were listed in “MD Anderson’s Pericardiocentesis Cardiac Catheterization Lab Registry.” The study was approved by MD Anderson’s Institutional Review Board.

### Patient Population

All patient data including imaging data was obtained using retrospective chart review. We collected patients’ demographic and clinical data, including age, sex, type of malignancy, prior cancer therapy (chemotherapy, immunotherapy, stem cell transplantation, surgery, and radiation), laboratory values, and cancer stage at the time of the index procedure. We also documented the clinical symptoms, signs, and echocardiographic findings of patients presenting with PE. An echo-free space 2 cm or larger was indicative of a large PE while echocardiographic evidence of tamponade was defined by presence of chamber collapse, mitral and tricuspid valve inflow variation on Doppler images, and inferior vena cava size and respiratory variation (6). Computed tomography scans and echocardiograms were reviewed to detect primary or metastatic tumors involving the heart and described as “cardiac involvement by primary tumor or metastases. The effusion pathology and microbiology results obtained at the time of PCC were also reviewed to determine the percentage of patients with ‘malignant effusion on cytology.” Cancer groups were divided into solid and hematological malignancies and then further sub-classified into 7 major types, including lung; breast; colon and other gastrointestinal malignancies (such as esophageal, stomach, hepatic, and pancreatic malignancies); renal and genitourinary malignancies; other solid tumors; lymphomas; and leukemia and other hematological malignancies. Patients’ cancers, were stratified as “advanced” (stage III or IV) or “non-advanced” (stage I or II). Determinants of recurrent PE requiring drainage were reviewed. A recurrent PE requiring drainage was defined as an effusion that caused clinical signs or symptoms as well as showed echocardiographic evidence of tamponade, and required drainage (either a pericardial window or repeated PCC). Patients who underwent a pericardial window for the index PE were excluded from the study.

### Pericardiocentesis Procedure

Patients underwent primary percutaneous PCC, which, for therapeutic and/or diagnostic purposes, was guided by echocardiography, computed tomography, fluoroscopy, or combined echocardiography and fluoroscopy in the cardiac catheterization laboratory. Percutaneous PCC was performed using either the subcostal or the lateral/intercostal approach, whichever provided the shortest distance from the skin to the pericardial cavity and preferably lateral in the thrombocytopenic patients (7, 8). A pericardial drain was placed in each patient and was removed once the amount of drainage was less than 30 cc in a 24-h period or if the duration of the drain placement exceeded 7 days. Handheld bedside echocardiography was encouraged immediately prior to drain removal, but the decision to use it was left up to the treating physician. As a routine practice, formal echocardiography was performed prior to drain removal as well as at follow-up in the outpatient cardiology clinic at 4–6 weeks and at 3–6 months to assess for PE recurrence. Procedure failure was defined as failure to place the catheter in the pericardial space or the presence of less than 10 ml drainage during the initial procedure. Procedure complications were defined as cardiac death, cardiac perforation, pneumothorax, or bleeding requiring transfusion during or within a few days after the procedure, after ruling out other obvious causes of such events.

### Data Analysis

Continuous variables were described as means ± standard deviations (SDs) or as medians with interquartile ranges (IQRs). Categorical variables were described as counts and percentages. The time to the first recurrent PE requiring drainage was defined as the time from the index PCC with pericardial drain placement to the first recurrent PE requiring drainage with either repeat PCC or a pericardial window. Patients without recurrent PE requiring drainage were censored at the time of death or last follow-up. The event of interest was recurrent PE requiring drainage with either repeat PCC or a pericardial window. Death without recurrent PE requiring drainage was considered as a competing risk event, an event that precludes the occurrence of the event of interest, recurrent PE (9). When a competing risk of death exists, it may not be appropriate to simply censor patients who died before they had a chance to experience recurrent PE. Ignoring the competing risk could result in incorrect estimation of the risk of recurrent PE. Therefore, univariate and multivariate Fine-Gray models were used to assess the covariates’ effects on the cumulative incidence of recurrent PE, accounting for death as a competing risk (10). Overall survival (OS) was defined as the time from the index PCC to death or last follow-up. Univariate and multivariate Cox regression models were used to identify risk factors associated with death. For model selection, the backward elimination method (for the Fine-Gray models) and stepwise selection method (for OS) were used. Subdistribution hazard ratios (sHRs) and 95% confidence intervals (CIs) were provided for Fine-Gray models and hazard ratios (HRs) and 95% CIs were provided for Cox regression models, as appropriate. *P*-values less than 0.05 were considered statistically significant. SAS version 9.4 (SAS Institute, Inc., Cary, North Carolina) was used for data analysis. Median follow up was determined using reverse Kaplan-Meier curve.

## Results

### Patients’ Baseline Characteristics

The cohort included 418 patients (mean age, 53 ± 16 years) with an index PCC. The patients’ baseline characteristics are summarized in Table 1.

**TABLE 1:** Baseline characteristics of the patients (*N* = 418).

Characteristic	Count
Age (years), mean ± SD	53 ± 16
Sex, n (%)	
Female	193 (46.2)
Male	225 (53.8)
Laboratory values	
Hemoglobin (g/dL), mean ± SD	10.11 ± 1.89
Platelets (k/mL), median (IQR)	211.5 (106–321)
International normalized ratio, mean (SD)	1.24 ± 0.25
Creatinine (mg/dL), median (IQR)	0.84 (0.65–1.13)
Malignancy stage, n (%)	
Unknown	1
Non-advanced (stage I or II)	9 (2.2)
Advanced (stage III or IV),	408 (97.8)
Approach for PCC, n (%)	
Subcostal	256 (61.2)
Intercostal	162 (38.8)
Echocardiographic evidence of tamponade at presentation, n (%)	398 (95.2)
Malignant effusion on cytology, n (%)	269 (64.4)
Anti-inflammatory medication use (colchicine, steroids, or NSAIDs),	49 (11.7)
Cardiac involvement by primary tumor or tumor metastases, n (%)	35 (8.4)
Cancer subgroup, n (%)	
Unknown	3
Hematologic	144 (34.7)
Solid	271 (65.3)
Cancer type, n (%)	
Unknown	5
Lymphoma	61 (14.7)
Lung	127 (30.6)
Breast	42 (10.1)
Colon and other GI	26 (6.3)
Kidney and GU	43 (10.4)
Leukemia and other hematological	83 (20)
Other solid cancers	33 (8)
Cancer treatment, n (%)	
Radiation	165 (39.5)
Chemotherapy	367 (87.8)
Surgery	126 (30.1)
Immunotherapy	91 (21.8)
Stem cell Transplant	63 (15.1)

*GI, gastrointestinal; GU, genitourinary; IQR, interquartile range; NSAID, non-steroidal anti-inflammatory drug; PCC, pericardiocentesis; SD, standard deviation.*

Most patients had advanced cancers (stage III or IV). All patients had large PEs, and most presented with echocardiographic evidence of tamponade (95%). Eight percent of patients had imaging evidence of cardiac metastasis. Two-thirds of the patients had malignant effusion on cytologic examination. Anti-inflammatory agents were prescribed in 11.8% of patients.

### Follow-Up and Outcomes

**FIGURE 1:**
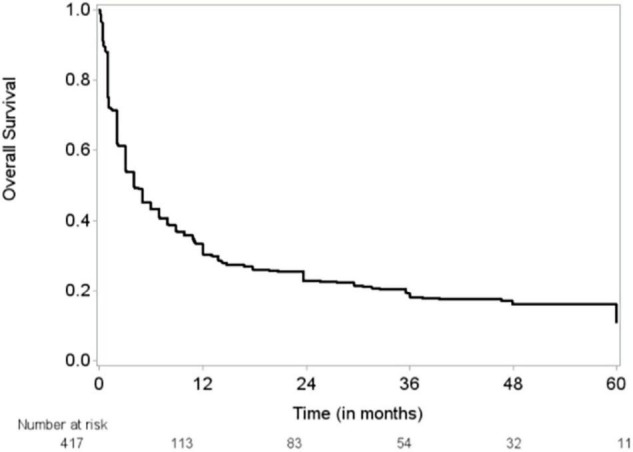
Kaplan-Meier curve for overall survival (OS). Outcome for 1 patient was missing.

**FIGURE 2:**
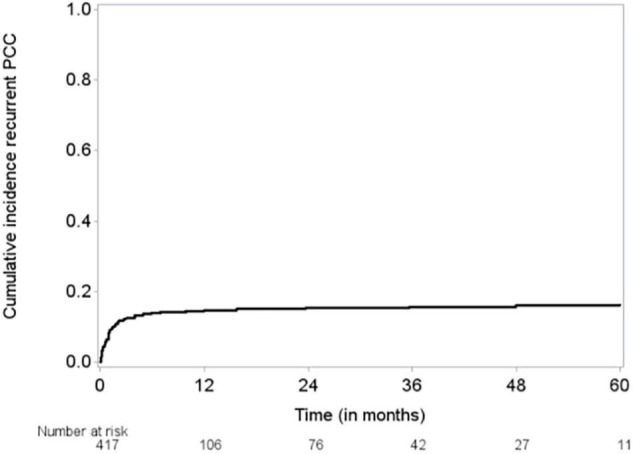
Cumulative incidence of recurrent pericardial effusion requiring drainage by Aalen-Johansen estimator. Outcome for 1 patient was missing.

**FIGURE 3:**
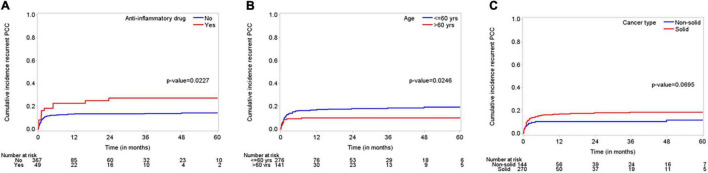
“Cumulative incidence plots” displaying incidence of recurrent effusions for subgroups including **(A)**; anti-inflammatory medications (use versus non-use), **(B)**; age (young between 18 and 60 years versus elderly > 60 years), and **(C)**; cancer type (solid versus non-solid tumors).

The median follow-up time was 48 months (95% CI, 43–51 months), and the median OS duration was 3.9 months (Figure 1). Majority of patients (92%) reported improvement in symptoms after draining pericardial effusion. Among all the patients who had index PCC, the rates of procedure complications and procedure failure were very low (0.24% each); the single procedure complication was a cardiac perforation (Table 2). Recurrent PE requiring drainage occurred in 65 (15.6%) patients; in 63 (15%) of these patients, it occurred within 1 year of the index PCC (Figure 2). Three hundred thirty-eight (80.9%) patients died by the end of the follow-up period. Cumulative incidence plots showed a statistically significant increase in recurrence of pericardial effusion in young patients and with anti-inflammatory medication use (Figure 3).

**TABLE 2:** Outcomes of patients with index pericardiocentesis.

Outcome	n (%)
Symptomatic improvement	385 (92)
Recurrent PE requiring drainage	65 (15.6)
Survival at end of follow-up	80 (19.1)
Procedure complications	1 (0.24)
Procedure failure	1 (0.24)

*PE: Pericardial Effusion.*

### Factors Determining Recurrence of Pericardial Effusion Requiring Drainage

The covariates that affect the incidence of recurrent PE requiring drainage are shown in Table 3. Univariate Fine-Gray models with death as a competing risk identified younger age, higher serum creatinine and hemoglobin levels, cardiac invasion by the tumor, and chemotherapy and surgery as the factors that have a significant increasing effect on the cumulative incidence of recurrent PE. The multivariate Fine-Gray model identified younger age, anti-inflammatory medication use, and solid malignancy as the factors with an increasing effect on the cumulative incidence of recurrent PE, while having echocardiographic evidence of tamponade at presentation and receiving immunotherapy were associated with a decreasing effect on the cumulative incidence of recurrent PE.

**TABLE 3:** Univariate and multivariate predictors of recurrent pericardial effusion requiring drainage.

	Univariate model		Multivariate model	
		
Covariate	sHR (95% CI)	*P*-value	sHR (95% CI)	*P*-value
Age (years)^1^	0.983 (0.968–0.998)	0.026	0.978 (0.960–0.997)	0.023
Sex				
Female	1.000			
Male	0.825 (0.508–1.339)	0.44		
Duration of drain placement^1^, days	0.818 (0.626–1.067)	0.14		
Hemoglobin (g/dL)^1^	1.178 (1.051–1.322)	0.005		
Platelets (k/mL)^1^	0.999 (0.998–1.000)	0.17		
International normalized ratio^1^	0.883 (0.364–2.144)	0.78		
Creatinine (mg/dL)^1^	1.017 (1.014–1.020)	< 0.0001		
Malignancy stage				
Non-Advanced				
Advanced (stage III or IV)	3.168 (0.460–400.356)	0.42		
Approach for pericardiocentesis				
Subcostal	1.000			
Intercostal	0.777 (0.467–1.292)	0.33		
Echocardiographic evidence of tamponade at presentation^2^	0.134 (0.083–0.216)	< 0.0001	0.154 (0.095–0.250)	< 0.0001
Malignant effusion on cytology^2^	1.171 (0.698–1.965)	0.55		
Anti-inflammatory medication use^2^ (Colchicine, steroids or NSAIDs)	2.015 (1.109–3.662)	0.022	1.897 (1.046–3.441)	0.035
Cardiac involvement by primary tumor or tumor metastases^2^	2.090 (1.083–4.035)	0.028		
Cancer treatment				
Radiation therapy^2^	1.507 (0.930–2.443)	0.10		
Chemotherapy^2^	4.514 (1.076–18.937)	0.039		
Surgery^2^	1.955 (1.204–3.177)	0.007		
Immunotherapy^2^	0.346 (0.148–0.811)	0.015	0.312 (0.135–0.719)	0.006
Stem cell transplant^2^	0.544 (0.238–1.246)	0.15		
Cancer subgroup				
Hematologic	1.000		1.000	
Solid	1.668 (0.947–2.938)	0.08	2.357 (1.243–4.467)	0.009
Cancer type				
Lymphoma	1.000			
Lung	1.175 (0.544–2.539)	0.68		
Breast	1.268 (0.499–3.222)	0.62		
Colon and other GI	1.583 (0.570–4.397)	0.38		
Kidney and GU	0.963 (0.341–2.723)	0.94		
Leukemia; other hematologic	0.554 (0.205–1.499)	0.25		
Other solid cancers	1.505 (0.554–4.089)	0.42		

*GI, gastrointestinal; GU, genitourinary; NSAID, non-steroidal anti-inflammatory drug; sHR, subdistribution hazard ratio (The sHR represents the ratio obtained from the Fine-Gray model with a competing risk of death).*

*^1^For this variable, the sHR is presented in 1-unit changes.*

*^2^For this variable, the sHR is presented considering “No” as a reference group.*

### Factors Determining Survival

The predictors of death are shown in Table 4. Univariate Cox regression models identified that older age, malignant effusion on cytological examination, and primary lung cancer were associated with an increased risk of death. Stem cell transplant and primary lymphoma were associated with a decreased risk of death. The multivariate Cox model identified that malignant effusion on cytological examination, not using anti-inflammatory agents, and non-lymphoma malignancies were associated with an increased risk of death.

**TABLE 4:** Univariate and multivariate predictors of all-cause mortality.

	Univariate model		Multivariate model	
		
Covariate	HR (95% CI)	*P*-value	HR (95% CI)	*P*-value
Age (years)^1^	1.009 (1.002–1.016)	0.010	1.007 (1.000–1.014)	0.0489
Sex				
Female	1.000			
Male	1.071 (0.864–1.328)	0.53		
Duration of drain placement^1^, days	0.987 (0.881–1.105)	0.82		
Hemoglobin (g/dL)^1^	0.961 (0.908–1.018)	0.18		
Platelets (k/mL)^1^	1.000 (0.999–1.000)	0.57		
International normalized ratio^1^	1.570 (0.987–2.500)	0.06		
Creatinine (mg/dL)^1^	1.002 (0.993–1.011)	0.65		
Malignancy stage				
Non-advanced	1.000			
Advanced (stage III or IV)	0.924 (0.458–1.864)	0.83		
Approach for pericardiocentesis				
Subcostal	1.000			
Intercostal	1.045 (0.840–1.300)	0.69		
Echocardiographic evidence of tamponade at presentation^2^	1.439 (0.869–2.383)	0.16		
Malignant effusion on cytology^2^	1.495 (1.188–1.881)	0.0006	1.286 (1.006–1.645)	0.04
Anti-inflammatory agents use^2^ (colchicine, steroids, or NSAIDs)	0.571 (0.398–0.819)	0.002	0.624 (0.426–0.916)	0.02
Cardiac involvement by primary^2^ tumor or tumor metastases	1.308 (0.912–1.875)	0.14		
Cancer treatment				
Radiation therapy^2^	1.155 (0.929–1.435)	0.19		
Chemotherapy^2^	1.100 (0.781–1.549)	0.59		
Immunotherapy^2^	1.175 (0.934–1.480)	0.17		
Surgery^2^	0.833 (0.638–1.088)	0.18		
Stem cell transplant^2^	0.730 (0.534–0.996)	0.047		
Cancer subgroup				
Hematologic	1.000			
Solid	1.600 (1.266–2.022)	< 0.0001		
Cancer type				
Lymphoma	1.000		1.000	
Lung	2.533 (1.725–3.718)	< 0.0001	2.387 (1.622–3.513)	< 0.0001
Breast	2.338 (1.474–3.709)	0.0003	2.028 (1.273–3.233)	0.0029
Colon and other GI	2.139 (1.262–3.626)	0.005	2.048 (1.208–3.472)	0.0078
Kidney or GU	2.650 (1.647–4.265)	< 0.0001	2.692 (1.670–4.338)	< 0.0001
Leukemia and other hematologic malignancies	1.927 (1.280–2.902)	0.002	1.902 (1.263–2.866)	0.0021
Other solid cancers	1.784 (1.079–2.951)	0.024	1.745 (1.052–2.894)	0.0310

*GI, gastrointestinal; GU, genitourinary; HR, hazard ratio; NSAID, non-steroidal anti-inflammatory drug.*

*^1^For this variable, the HR is presented in 1-unit changes.*

*^2^For this variable, the HR is presented considering “No” as a reference group.*

## Discussion

In this study, we report on a cohort of 418 cancer patients presenting with PE treated with percutaneous PCC. Our study had several key findings. First, factors independently associated with an increasing effect on the cumulative incidence of recurrent PE requiring drainage included younger age, anti-inflammatory medication use, and solid tumors, whereas factors associated with a decreasing effect on the cumulative incidence of recurrent PE requiring drainage included having echocardiographic evidence of tamponade at presentation and receiving immunotherapy. Second, factors independently associated with poor OS included older age, malignant effusion on cytology, non-use of anti-inflammatory agents, non-lymphoma cancers and primary lung cancer. Third, PEs can be successfully drained with a very low rate of complications. Lastly, only 16% of patients presented with recurrent PEs requiring drainage, and almost all occurred within the first year after the index PCC.

In our study, the most frequent tumors associated with PEs requiring drainage were lung cancers (30.4%), followed by lymphomas (14.6%), leukemias (13.4%), and breast cancers (10.3%). While some studies have reported a similar percentage of cancer patients with pericardial effusion having hematological malignancies (1), others have reported a relatively less prevalence (4). Since most of these studies have been single center, this difference in observation can be explained by different patient population in each center. Most patients had advanced malignancies. In about two-thirds of our patients, cytological analysis of the pericardial fluid was positive for malignant cells; this was an independent predictor of a poor prognosis. This finding is in line with previous studies showing that recurrent, malignant PE occurs more commonly in patients with previously identified cardiac involvement than in those without it (11–14). In our study, 95% patients had echocardiographic evidence of tamponade while 5% patient underwent PCC for various reasons including clinical signs and symptoms related to large pericardial effusion, to establish the diagnosis of cancer, and for cancer staging.

In a retrospective analysis of cancer patients whose cumulative incidence of recurrent PE was 26.1% at 2-year interval from their index PCC, the use of anti-inflammatory agents was linked to a lower rate of death and PE recurrence (15). Similarly, we found that not using anti-inflammatory agents was associated with poor OS. However, in contrast to that study, we found that the risk of recurrence was higher with the use of these agents. This may represent a selection bias for the use of such therapies in patients who are generally thought to have a higher risk of recurrence or may reflect the use of anti-inflammatory agents to facilitate pericardial drain removal in patients requiring longer periods of pericardial drainage. This selection bias can also be due to use of such agents in patients with progressive primary cancer with increased tendency to develop recurrent effusions. Also, using anti-inflammatory agents in patients with hemorrhagic effusion can potentially lead to increased bleeding in pericardial space and increased recurrence risk (16, 17). In another study, rate of recurrence of pericardial effusion requiring pericardiocentesis was reduced from 23 to 11% with catheter drainage for 3-5 days as compared to not using an indwelling catheter (1). This alone was sufficient to reduce the risk of recurrence considerably and our data suggests that use of anti-inflammatory medications in the immediate peri-procedure phase (first week) might be hindering the beneficial effect of extended catheter drainage and mechanically induced adhesions.

Previous studies have shown conflicting evidence regarding the association between malignant cells in the pericardial fluid and poor outcomes in cancer patients (18–20). Our results indicate an association between malignant cells in the pericardial fluid and worse OS in cancer patients. Results from our analysis showed that patients with solid tumors had poor survival if they had malignant cells in the pericardial fluid; however, the outcomes did not differ for other cancer types when stratified by the results of cytological analysis.

In our study, almost all patients who developed recurrent PE developed it within 1 year after the index PCC. Specifically, the PE recurrence rate was only 15% in the first year after the index PCC and increased to only 16% after 5 years. This rate is lower than that reported previously (13). The low recurrence rate in our study can be explained by the close monitoring of the pericardial drain output, the standardized approach used for drain removal (based on 24-h drain output), and the encouraged use of echocardiography prior to drain removal. Among the patients who had recurrent PEs requiring drainage, the OS and recurrence rates did not differ between the patients who had had a pericardial window versus those who were treated percutaneously. This finding establishes the safety and utility of PCC in high-risk patients with cancer who present with PE recurrence (21, 22).

No randomized studies have compared the percutaneous drainage of PEs to the surgical drainage of PEs. Retrospective studies have shown that surgical drainage can reduce recurrence but increase the risk of peri-procedure complications (23). The American Heart Association and American College of Cardiology offer no guidelines on the management of pericardial disease. According to the 2015 European Society of Cardiology guidelines for pericardial disease, the treatment of cardiac tamponade related to a malignant PE effusion is a class I indication for PCC. Surgical pericardiotomy is indicated when PCC cannot be performed (class IIa; level of evidence B), but the surgical procedure may be associated with a higher rate of complications than PCC is and may not result in better outcomes (24). Guidelines for the treatment of PE recurrence do not exist. The choice between catheter-based and surgical drainage is usually made by a multidisciplinary team that includes the patient’s oncologist, cardiologist, and thoracic surgical team, and it should be individualized to each patient and consider the patient’s preference. In both percutaneous and PE treatment, the subcostal and intercostal (apical/lateral) approaches are similarly efficacious. Whether these approaches are successful depends primarily on the characteristics and location of the effusion, the stability of the patient, and various laboratory and clinical characteristics, including the presence of a chest wall tumor, the patient’s history of chest wall radiation or abdominal surgery, or an apical loculation of the pericardial effusion (25–27). Our study found no difference in survival or PE recurrence between the 2 percutaneous approaches.

The safety of PCC was well demonstrated in our study in which the rates of procedure failure and complications were very low (0.24% each). One systematic review showed that the incidences of recurrent PE after isolated pericardiocentesis, PCC with extended catheter drainage, pericardial sclerosis, and percutaneous balloon pericardiotomy were 38.3%, 12.1%, 10.8%, and 10.3%, respectively (14). Despite being associated with a relatively high rate of recurrence, PCC continues to be a very attractive option for high-risk cancer patients. Some prefer to use surgical pericardial windows rather than PCC as the initial PE treatment in cancer patients owing to the high rate of cancer invasion into the pericardium and the high recurrence rate of PE; however, pericardial windows may be suboptimal for these patients because such patients tend to be frail and because the use of pericardial windows may delay their recovery from surgery and general anesthesia and thus affect their cancer treatment schedule. The high success rate of PCC and its low complication and recurrence rates in our large cohort of cancer patients shows the value of the percutaneous procedure, with continued drainage over a few days, as a first line therapy for large PEs in these patients. That PCC is associated with no significant delay in cancer treatment (surgery, chemotherapy, immunotherapy, or radiation therapy) further supports its use in this population (28).

As suggested in our study, routine surveillance echocardiograms done at 3–6 weeks and at 4–6 months after index PCC can help determine which patients are more likely to develop recurrent effusions and may warrant closer monitoring and subsequent surveillance echocardiograms. In the current study, the median OS duration for cancer patients requiring PCC was 3.9 months (95% CI, 3–4.9 months). Although this duration is a little higher than that reported previously (15), the finding reiterates that PE requiring drainage is a poor prognostic marker in patients with cancer because it is indicative of advanced malignancy.

### Study Limitations

Because this study was a retrospective chart review, it was subject to selection bias, as decisions regarding the procedure, entry site, imaging guidance, drainage duration, and use of anti-inflammatory agents were individualized to each patient and at the discretion of the treating physician. The use of standardized protocols for PCC at our institution as well as protocols for surveillance imaging prior to and after drain removal may have helped counter the bias to some extent. Initial performance status data were not obtained, and symptomatic improvement and quality-of-life metrics were not quantified or collected owing to the urgent/emergent nature of the procedure, though immediate symptom relief was often recognized. Outcomes of patients with recurrent pericardial effusion managed with therapies such as pericardial window or instillation of intra-pericardial sclerosing agents were not included in our study. Since this study included patients from ‘Cardiac Catheterization Lab’ database, a direct comparison cannot be made with patients who had malignancy but did not meet inclusion criteria for the study and hence did not undergo PCC.

## Conclusion

Pericardiocentesis is an attractive option in cancer patients with large pericardial effusion with acceptable recurrence rate. Aggressive cancers (younger patients with solid malignancy) have an increased risk of recurrent PE within the first year from the initial PCC, while elderly patients with lung cancer and malignant PE cytology have worse survival. Cancer patients requiring treatment with immunotherapy appear less likely to require additional PCC. Future studies will continue to refine and align cancer and cardiovascular care to benefit patients facing this double jeopardy.

## Data Availability Statement

The raw data supporting the conclusions of this article will be made available by the authors, without undue reservation.

## Ethics Statement

The studies involving human participants were reviewed and approved by MD Anderson Institutional Review Board. The ethics committee waived the requirement of written informed consent for participation.

## Author Contributions

TA, CI, AD, and EM contributed to the conception and design of the study. JS, EK, and NP organized the database. JS performed the statistical analysis. TA, JL-M, and PK wrote the first draft of the manuscript. SY, SH, MC, KM, AV, AD, CI, and SS wrote sections of the manuscript. All authors contributed to manuscript revision, read, and approved the submitted version.

## Conflict of Interest

The authors declare that the research was conducted in the absence of any commercial or financial relationships that could be construed as a potential conflict of interest.

## Publisher’s Note

All claims expressed in this article are solely those of the authors and do not necessarily represent those of their affiliated organizations, or those of the publisher, the editors and the reviewers. Any product that may be evaluated in this article, or claim that may be made by its manufacturer, is not guaranteed or endorsed by the publisher.
